# Collapsing Focal Segmental Glomerulosclerosis in Siblings With Compound Heterozygous Variants in *NUP93* Expand the Spectrum of Kidney Phenotypes Associated With Nucleoporin Gene Mutations

**DOI:** 10.3389/fped.2022.915174

**Published:** 2022-07-07

**Authors:** Rachel K. Cason, Anna Williams, Megan Chryst-Stangl, Guanghong Wu, Kinsie Huggins, Kaye E. Brathwaite, Brandon M. Lane, Larry A. Greenbaum, Vivette D. D’Agati, Rasheed A. Gbadegesin

**Affiliations:** ^1^Division of Nephrology, Department of Pediatrics, Duke University Medical Center, Durham, NC, United States; ^2^Division of Pediatric Nephrology, Children’s Hospital at Montefiore, The Bronx, NY, United States; ^3^Division of Pediatric Nephrology, Emory University School of Medicine and Children’s Healthcare of Atlanta, Atlanta, GA, United States; ^4^Department of Pathology and Cell Biology, Columbia University Medical Center, New York, NY, United States

**Keywords:** nephrotic syndrome, focal segmental glomerulosclerosis, collapsing FSGS, nucleoporin genes, *APOL1*

## Abstract

**Background:**

Focal segmental glomerulosclerosis (FSGS) is a major cause of end stage kidney disease, with the collapsing form having the worst prognosis. Study of families with hereditary FSGS has provided insight into disease mechanisms.

**Methods:**

In this report, we describe a sibling pair with *NUP93* mutations and collapsing FSGS (cFSGS). For each brother, we performed next generation sequencing and segregation analysis by direct sequencing. To determine if the variants found in the index family are a common cause of cFSGS, we screened 7 patients with cFSGS, gleaned from our cohort of 200 patients with FSGS, for variants in *NUP93* as well as for *APOL1* high-risk genotypes.

**Results:**

We identified segregating compound heterozygous *NUP93* variants *(1) c.1772G* > *T p.G591V, 2) c.2084T* > *C p.L695S)* in the two brothers. We did not find any pathogenic variants in the seven patients with cFSGS from our cohort, and as expected five of these seven patients carried the *APOL1* high-risk genotype.

**Conclusion:**

To the best of our knowledge, this is the first report of cFSGS in patients with *NUP93* mutations, based on this report, mutations in *NUP93* and other nucleoporin genes should be considered when evaluating a child with familial cFSGS. Determining the mechanisms by which these variants cause cFSGS may provide insight into the pathogenesis of the more common primary and virus-mediated forms of cFSGS.

## Introduction

Nephrotic syndrome (NS) is a major cause of chronic kidney disease (CKD) in children, and it is the most common glomerular disease encountered in children with an overall incidence of 2.92 per 100,000 children (1). Steroid Resistant NS (SRNS), defined as failure to achieve remission following 4–6 weeks of standard corticosteroid therapy, is seen in about 20% of all cases of childhood NS (2). SRNS is the most severe form of childhood NS, and it is a major cause of end stage kidney disease (ESKD) in children. The cause of SRNS is often unknown; however genetic studies have shown that 10–30% of SRNS cases are monogenic disease due to pathogenic variants in one of more than 60 genes (3). All of these causative genes are expressed in the podocyte, leading to podocyte dysfunction; hence the term “podocytopathy” has been applied to these disorders.

Nucleoporins (Nups) are proteins associated with the nuclear pore complex (NPC) in a variety of kidney cells. They are important in cellular molecular trafficking of proteins, RNA, and other molecules between the cytoplasm and the nucleoplasm (4). Pathogenic variants in *NUP* genes have been reported as causes of SRNS, with histologic changes including minimal change disease (MCD) and focal segmental glomerulosclerosis (FSGS) (4). While pathogenic variants have been reported to cause different morphologic types of FSGS, to the best of our knowledge, mutations in *NUP93* and other nucleoporin genes have not been reported previously as a cause of collapsing FSGS (cFSGS) (5). Other genes that have been identified as cause of cFSGS include *ACTN4, COQ2, SCARB2*, and *TRPC6* (6–9). Collapsing FSGS is characterized by retraction and collapse of the glomerular capillaries associated with hypertrophy and hyperplasia of the overlying glomerular epithelial cells; it portends the worst prognosis among histologic subtypes of FSGS (10, 11). This subtype was first described in patients with HIV-associated nephropathy and has been seen in association with other viral infections such as SARS-CoV-2, parvovirus B19 and cytomegalovirus (12–15). cFSGS has also been documented to occur after treatment with certain medications, such as bisphosphonates and interferon, and in association with systemic diseases, such as systemic lupus erythematosus and thrombotic microangiopathy (16–19). Here, we have identified a white sibling pair with cFSGS due to compound heterozygous mutations in *NUP93*. We describe their clinical course and screen other patients with cFSGS in our cohort for these variants. We also determine the impact of the pathogenic variants on the three-dimensional (3D) structure of the nucleoporin 93 (NUP93) protein and the potential implications of the changes for the more common primary and infection-mediated cFSGS.

## Materials and Methods

### Clinical Data

The Institutional Review Board of the Duke University Medical Center (Durham, NC, United States) approved the study. Written informed consent/assent was obtained from all participants prior to inclusion in the study. The methods of subject recruitment have been previously reported (15). Inclusion criteria for the study were (1) proteinuria, (2) biopsy proven MCD or FSGS. Clinical data obtained included family history, age at onset of disease, age at end stage kidney disease, age at kidney transplantation and history of disease recurrence following kidney transplantation.

### Next Generation Sequencing and 3D Variant Modeling

We obtained DNA from the affected brothers and their unaffected parents and performed whole genome sequencing in the two affected siblings. Whole genome sequencing was performed by Veritas Genetics (Danvers, MA). Samples were sequenced on the HiSeq X with a 2 × 150 bp paired-end sequencing configuration. Variant calling, annotation, and variants filtering were carried out using previously published standard methods (15). All variants of interest including *APOL1* G0, G1, and G2 were confirmed by direct sequencing. *In silico* 3D modeling of NUP93 peptides was carried out as previously described (15).

### Case Descriptions and Results

**FIGURE 1:**
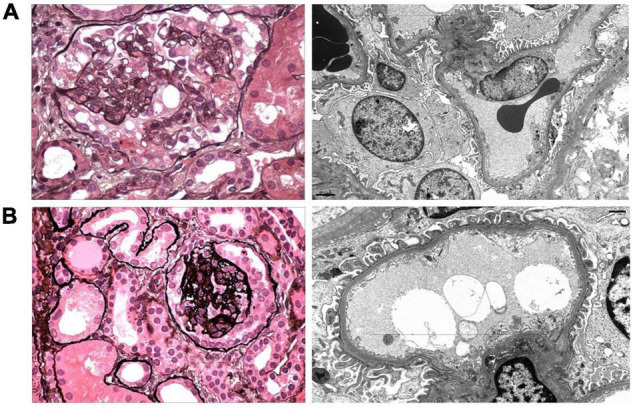
Representative images of the kidney biopsies from Patient 1 **(A)** and Patient 2 **(B)** are shown. **(A)** A representative glomerulus has segmental collapsing sclerosis with implosive wrinkling and collapse of glomerular capillary walls and hypertrophy and hyperplasia of the overlying glomerular epithelial cells (Jones methenamine silver, ×600). Electron microscopy shows irregular (40%) foot process effacement with swollen podocyte cell bodies and open-appearing cytoplasm with reduced organellar content (× 6,000). **(B)** There is focal global tuft collapse with a corona of hyperplastic glomerular epithelial cells and focal podocyte shedding and apoptosis (Jones methenamine silver, ×600). Electron microscopy reveals mild (25%) foot process effacement (×10,000).

**Patient 1:** A 5-year-old white male of European descent presented with asymptomatic proteinuria. He was noted to have nephrotic range proteinuria (urine protein/creatinine ratio 17 mg/mg). He was unresponsive to 6 weeks of corticosteroid therapy and kidney biopsy showed cFSGS (Figure 1A). He had negative testing for HIV and parvovirus B19. He was treated with the calcineurin inhibitor (CNI) cyclosporine for 3 months before progressing to end stage kidney disease (ESKD) at the age of 6 years. He received a living unrelated kidney transplantation at the age of 7 years. He did not develop recurrence of FSGS in his kidney transplant as demonstrated on two kidney allograft biopsies. He continued to have a functioning kidney allograft after more than 10 years of follow-up. The patient’s clinical characteristics are shown in Table 1.

**TABLE 1:** Clinical characteristics of siblings with *NUP93* mutations.

	Patient 1	Patient 2
Age at diagnosis (years)	5	2
Response to corticosteroid (Yes/No/NA)	No	NA
Response to calcineurin inhibitors (Yes/No/NA)	No	NA
Age at ESKD (years)	6	10
Pre-transplant peak Blood Urea Nitrogen (BUN) (mg/dl)	95	69
Pre-transplant peak Creatinine (mg/dl)	18.1	17.7
Pre-transplant peak protein: creatinine ratio (mg/mg)	17.5	11.7
Age at transplant (years)	7	10
cFSGS recurrence (Yes/No)	No	No
Age at last follow up (years)	18	14
Current creatinine (mg/dl)	1.4	0.8

*NA, Not applicable; cFSGS, collapsing focal segmental glomerulosclerosis.*

**Patient 2:** Patient 2 is the younger brother of Patient 1. He was screened for proteinuria at the age of 2 years because of history of FSGS in his older brother, and he was found to have asymptomatic nephrotic range proteinuria (urine protein/creatinine ratio of 3.2 mg/mg). Kidney biopsy showed collapsing FSGS (Figure 1B). He had negative testing for HIV as part of his transplant evaluation. He was treated with angiotensin converting enzyme inhibitor alone and did not receive immunomodulating agents because of suspicion for monogenic SRNS. He developed ESKD at the age of 10 years and underwent living related kidney transplantation from a maternal uncle shortly after developing ESKD. The donor was not screened for mutations in *NUP93* since *NUP93* had not been identified at that time as a cause of monogenic SRNS (both brothers had extensive genetic testing, but it did not include *NUP93*). He did not develop recurrence of FSGS in his kidney transplant as evidenced by four transplant kidney biopsies. He had normal kidney function and no proteinuria after 4 years of follow-up. Clinical characteristics for Patient 2 are shown in Table 1.

### Kidney Biopsy

**Patient 1:** (Figure 1A) Kidney biopsy was performed at age 5 years. Among 33 glomeruli present, 13 had global glomerulosclerosis and 9 showed FSGS with predominantly collapsing features. Moderate tubular atrophy and interstitial fibrosis involved approximately 35% of the cortex. Glomerular tuft staining for IgM, C3, and C1q was observed in areas of sclerosis, consistent with non-specific trapping. Foot process effacement affected approximately 40% of the glomerular capillary surface area. The podocyte cell bodies appeared swollen with reduced organellar content.

**Patient 2:** (Figure 1B) Kidney biopsy was performed at age 2 years. Among 28 glomeruli sampled, 2 displayed global collapsing sclerosis with podocyte capping, hyperplasia, and focal apoptosis. Four glomeruli contained segmental lesions of sclerosis, 2 of which exhibited collapsing features. Podocytes of the remaining glomeruli appeared swollen. Tubular atrophy and interstitial fibrosis occupied 10% of the cortex, with prominent proximal tubular protein and lipid resorption droplets and early tubular microcyst formation. Sclerosing glomeruli contained deposits of IgM, C3, and C1q, with podocyte droplet staining for albumin. Foot process effacement involved approximately 25% of the glomerular capillary surface area.

### Whole Genome Sequencing

**FIGURE 2:**
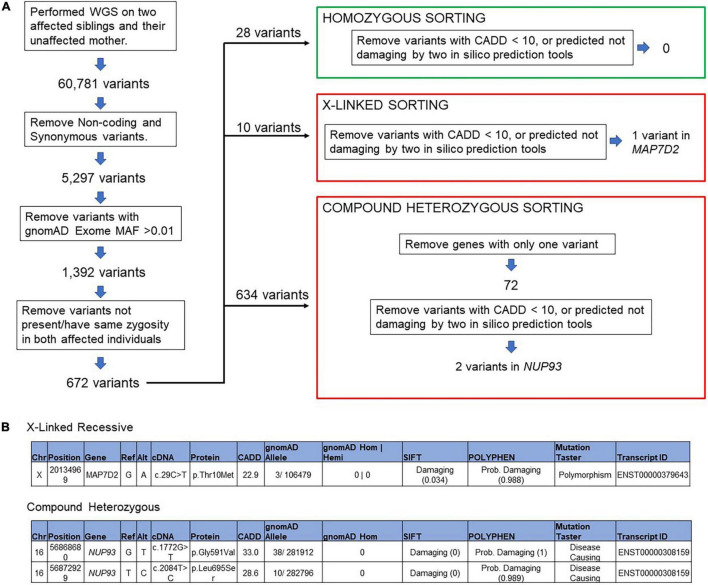
Filtering algorithm **(A)** variant filtering of the whole genome sequencing data in patient with collapsing FSGS, **(B)** segregating variants of unknown significant was identified in *MAP7D2* and a compound heterozygous variant in *NUP93.*

**FIGURE 3:**
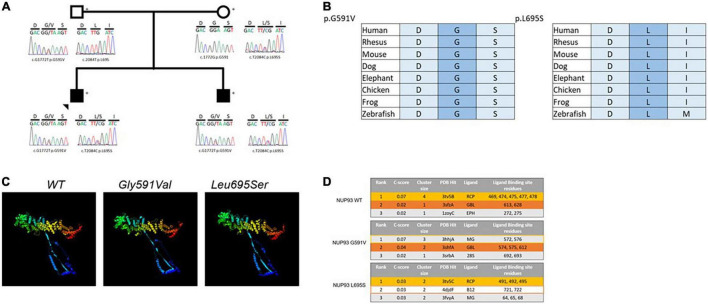
**(A)** Two rare variants in *NUP93* gene segregate with disease in two brothers with cFSGS, **(B)** the two variants are conserved in evolution down to zebrafish, **(C)** the 3D structures for the wild type (WT) NUP93 protein (left), the G591V variant (middle) and the L695S variant (right) showing structural changes in the amino terminus (blue) to the carboxyl terminus (red) in the two variants compared with the WT, **(D)** the top predicted ligands for WT NUP93 (top), G591V (middle) and L695S (bottom). The top two predicted ligands for WT NUP93, Acetyl-coenzyme A carboxylase (RCP, yellow) and Apaf-1 (GBL, orange) are disrupted in the G591V variant and L695AS variant, respectively.

We applied our standard filtering algorithm (Figure 2) (20) and identified segregating compound heterozygous variants (1) c.1772G > T p.G591V, (2) c.2084T > C p.L695S in *NUP93* in the two brothers (Figure 3A). Both variants, which occur in evolutionarily conserved amino acid residues, are rare with a minor allele frequency of < 0.00015 in the gnomAD database. Neither variant is present in biallelic (homozygous) state in any public database and both are predicted to be damaging by four *in silico* analysis tools (Figure 2). In addition we identified a variant in the gene *MAP7D2* c.29C > T p.Thr10Met. *MAP7D2* encodes for MAP7 domain containing 2 (Figure 2). Variants in *MAP7D2* have not been previously reported as a cause of nephrotic syndrome and the function of the gene is unknown.

### Three-Dimensional Modeling of Consequences of Variants on Nucleoporin 93 Protein

The variants are located in the carboxyl terminus of the NUP93 protein that is essential for nuclear pore formation. Additionally, *in silico* 3D modeling predicts that both variants will create structural alterations throughout the protein including the amino and the carboxyl terminal residues that will affect NUP93 ligand binding (Figures 3B–D).

### Screening of Patients With Collapsing FSGS for Variants in *NUP93* and *APOL1*

In our cohort of over 200 patients with biopsy proven FSGS, we identified seven children from six families with cFSGS. The clinical characteristics of these seven children are shown in Table 2. To determine whether the variants we found in the index family and *NUP93* variants are common cause of monogenic cFSGS, we screened these seven individuals for variants in the coding exons of *NUP93* and did not find any patient with biallelic pathogenic variants in *NUP93*. Since six of the seven individuals are of African ancestry, we screened all seven patients for the high risk *APOL1* genotype associated with CKD (21). Five of the seven individuals with cFSGS were found to have *APOL1* high-risk genotypes G1/G1, or G1/G2. The other two had the G0/G0 *APOL1* genotype. Two of the patients with the high-risk *APOL1* genotype also had parvovirus B19 infection at the time of presentation. As expected, the two index patients with the *NUP93* pathogenic variants had G0/G0 genotype for *APOL1*.

**TABLE 2:** *APOL1* and *NUP93* genotype in seven children with *cFSGS*.

Patient id	Sex	Age at onset (years)	Race	History of infection Y/N (Type)	*APOL1* genotypes	cFSGS *NUP93* genotype mutant/normal
6721	M	1.5	White	N	G0/G0	WT/WT
34337	M	17	Black	N	G1/G1	WT/WT
34178-1	M	11	Black	Y (parvovirus)	G1/G2	WT/WT
34178-100	M	11	Black	Y (parvovirus)	G1/G2	WT/WT
40674	M	10	Black	N	G1/G2	WT/WT
6671	F	8	Black	N	G0/G0	WT/WT
34384	F	Unknown	Black	N	G1/G2	WT/WT

*cFSGS, collapsing focal segmental glomerulosclerosis.*

## Discussion

Pathogenic variants in *NUP93* have been reported in association with MCD and various morphologic subtypes of FSGS. To the best of our knowledge, cFSGS has not been previously reported in patients with pathogenic *NUP93* variants; hence, our report expands the kidney phenotype associated with *NUP93* mutations. The reason for our findings is unclear but since other *NUP93* pathogenic variants have been associated with other types of FSGS and MCD, our observation is likely due to biallelic heterogeneity but we cannot rule out completely variable expressivity.

Nucleoporins have a critical role in regulating the transfer of signaling molecules between the cell nucleus and cytoplasm. *NUP93* encodes a central component of the NUP93 complex of proteins that serves as a major structural backbone of the nuclear pore complex (NPC). The compound heterozygous variants found in this family are located in the carboxyl terminus of the NUP93 protein, a region often associated with both kidney and non-kidney disease-causing variants. This area of the protein is critical for assembly of the NUP93 complex as well as nuclear pore formation (22). Both the G591V and L695S variants are located in binding regions for another component of the NUP93 complex, NUP155, suggesting that formation of this biologically important complex may be disrupted in patients carrying both mutant alleles (23). Additionally, 3D modeling analysis predicts that these variants will result in disruption of NUP93 binding to key nucleoporin-related molecules. The G591V variant is expected to alter binding to acetyl-coenzyme A carboxylase, the most likely predicted ligand of NUP93 and a regulator of long chain fatty acid composition of the nuclear pore-membrane interface (24). The NUP93 ligand with the second highest prediction score, apoptotic protease activating factor 1 (Apaf-1), is expected to be disrupted by L695S. Apaf-1 is known to associate with nucleoporins during translocation between the cytoplasm and nucleus as it serves roles in apoptosis signaling as well as the DNA damage response (25). It is not currently known if these specific altered ligand interactions contribute to the pathogenesis of *NUP93-*mediated disease, but the use of predictive modeling has uncovered new potential mechanisms that warrant further investigation. Both the G591V and L695S variants have been reported as homozygous variants or as compound heterozygous variants in trans with other variants, but inheritance of both the G591V and L695S variants in trans with each other has not been previously reported (5). All the previous reports of patients with these variants have described non-collapsing FSGS or MCD phenotype on kidney biopsy (4, 26, 27).

Collapsing FSGS has been reported in patients with a variety of infections, including HIV, parvovirus B19, and more recently SARS-CoV-2 (12, 13, 28). It is also associated with use of medications like interferon-alpha, -beta, or -gamma and the bisphosphonate pamidronate (16, 17). Most patients with cFSGS secondary to infections or medication exposure are of African ancestry and carry high-risk *APOL1* genotype (29). The index patients in this study did not have clinical evidence of any of these viral infections and they were not exposed to any of the medications associated with cFSGS. Also, they do not carry the high-risk genotype (homozygous G1 or G2 or compound heterozygous G1/G2) for *APOL1* genes. To determine if the compound heterozygous variants identified in this family are a common cause of monogenic familial and sporadic cFSGS, we screened other patients with cFSGS in our cohort and did not identify any patients with this combination of variants, suggesting that these compound heterozygous variants are unique to this family. However, we confirmed that *APOL1* high risk genotype is the most common risk factor for non-HIV associated cFSGS in our cohort, and our results confirmed previous reports by other Investigators (30). It is interesting to note that some patients with variants in *NUP93* have been reported to have recurrence of FSGS following kidney transplantation, suggesting that mutations in *NUP93* may cause disease by modulation of the immune cells (26). Our patients have been followed for 4–10 years after transplantation. They have had repeated kidney biopsies during the follow up and neither of them has been diagnosed with disease recurrence. In addition, both patients have functioning grafts and no proteinuria at the last follow-up.

## Conclusion

We report a family with cFSGS due to compound heterozygous mutation in *NUP93*, thereby expanding the spectrum of kidney phenotypes associated with genetic defects in *NUP93.* As the morphogenesis of cFSGS is postulated to involve rapid rates of podocyte depletion, studies to determine the cellular mechanisms by which these variants cause cFSGS may provide new insights into the pathogenesis of the more common primary and virus-mediated forms of cFSGS.

## Data Availability Statement

The datasets for this article are not publicly available due to concerns regarding participant/patient anonymity. Requests to access the datasets should be directed to the corresponding author.

## Ethics Statement

The studies involving human participants were reviewed and approved by the Duke University IRB. Written informed consent was obtained from the individual(s) next of kin for the publication of any potentially identifiable images or data included in this article.

## Author Contributions

RG designed the experiments. RG, RC, KB, AW, KH, BL, LG, and VD’A wrote the manuscript. RG, RC, AW, MC-S, KH, GW, and LG performed subjects’ enrollment and sample acquisition. MC-S, BL, KH RC, and RG carried out sequencing, analysis of sequencing data and *in silico* modeling. VD’A carried out renal histology and electron microscopy reading and interpretation. All authors reviewed and edited the manuscript.

## Conflict of Interest

The authors declare that the research was conducted in the absence of any commercial or financial relationships that could be construed as a potential conflict of interest.

## Publisher’s Note

All claims expressed in this article are solely those of the authors and do not necessarily represent those of their affiliated organizations, or those of the publisher, the editors and the reviewers. Any product that may be evaluated in this article, or claim that may be made by its manufacturer, is not guaranteed or endorsed by the publisher.

## Abbreviations

FSGS: Focal segmental glomerulosclerosis
cFSGS: collapsing FSGS
NS: Nephrotic syndrome
SRNS: Steroid Resistant Nephrotic Syndrome
CKD: chronic kidney disease
ESKD: end stage kidney disease
NUPs: Nucleoporins
NPC: Nuclear Pore Complex
MCD: Minimal Change Disease
CNI: calcineurin inhibitor
Apaf-1: apoptotic protease activating factor 1.
